# Supplementary material for: Deregulation of calcium homeostasis in Bcr-Abl-dependent chronic myeloid leukemia
Source: Oncotarget. 2018 May 29;9(41):26309–27. doi: 10.18632/oncotarget.25241 (PMC5995172; doi:10.18632/oncotarget.25241)
Supplement: Supplementary file 1 [file oncotarget-09-26309-s001.pdf]

Deregulation of calcium homeostasis in Bcr-Abl-dependent chronic myeloid leukemia

SUPPLEMENTARY MATERIALS

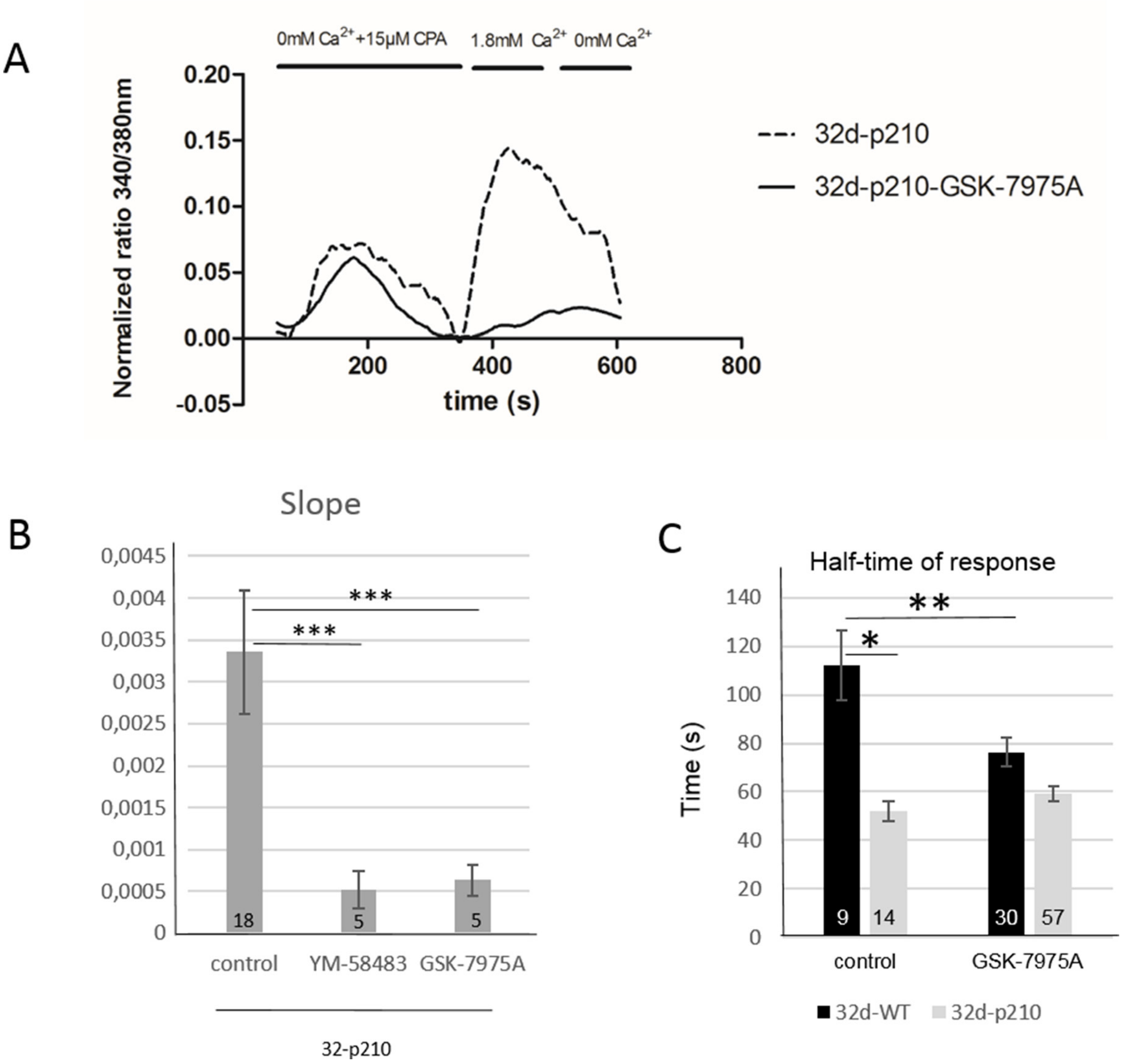

Supplementary Figure 1: Impact of Orai1 inhibitor, GSK-7975A in calcium entries. (**A**) Store-operated Ca^{2+} entry in 32d-p210 cell line in control condition (dotted line) and in presence of 1 µM GSK-7975A (dark line). The ER depletion was obtained by perfusion of 15 µM CPA (SERCA inhibitor) in 0 mM Ca^{2+} solution, which allowed SOCE recording in presence of 1.8 mM Ca^{2} prior to a 0 mM Ca^{2+} buffer incubation. (**B**) SOCE measurements (initial slope of intracellular Ca^{2+} rise) in p210-32d cells in control condition or after incubation of 10 µM YM 58483 or 1 µM GSK-7975A for 30 min. This experiment was done also with 10 µM GSK-7975A but no calcium entries could be measured showing a total SOCE inhibition in this experimental condition. (**C**) Quantification of half-time response of thrombin-induced Ca^{2+} transient with or without pre-incubation with 10 µM GSK-7975A during 30 minutes and incubated with 1 U/ml thrombin in 1.8 mM Ca^{2+} buffer in WT and 32d-p210 cells. The duration of the thrombin-induced Ca^{2+} transient is more dependent on SOCE in WT cells than in 32d-p210 cells. Bar graphs represent mean rates ± SEM. $^{*}P < 0.01$; $^{**}P < 0.005$; $^{***}P < 0.001$.
